# The effectiveness of COVID-19 Heterologous Vaccination: the experience from a Regional Hospital in Cameroon

**DOI:** 10.4314/ahs.v24i4.11

**Published:** 2024-12

**Authors:** Princewill Kum Unji, Alex Mambap Tatang, Samuel Angwafor, Loveline Lum Niba, Adji Minette Jaqueline Porro, Denis Nsame Nforniwe, Andreas Chiabi

**Affiliations:** 1 University of Bamenda, Clinical Sciences; 2 Bamenda Provincial Hospital

**Keywords:** COVID-19 vaccine, heterologous vaccination, homologous vaccination

## Abstract

**Background:**

With most COVID-19 vaccines requiring at least 2 doses, heterologous vaccination will facilitate vaccination programs where vaccine supplies fluctuate. However, with insufficient data on effects of heterologous vaccination in our setting, people remain reluctant to mix.

**Objectives:**

We seek to assess the effects of heterologous vaccination on morbidity and mortality.

**Methods:**

This was a 7 months retrospective study for COVID-19 patients managed by the Bamenda Regional Hospital, Cameroon, running from the 18th August 2021 to 28^th^ February 2022. Logistic regression used to asses relationship between predictors and outcome.

**Results:**

Our 1254 participants had a mean age of 50.1(±19.6) years, we had 24 (1.9%) being fully vaccinated, where 7 (29.2%) took heterologous vaccines. Also, 721 (57.5%) had the moderate/severe form of the disease. Those fully vaccinated had lower odds of having severe disease (p=0.037). However, heterologous vaccination compared to homologous vaccination had no significant difference on morbidity (p=0.729). Among patients who died, none was fully vaccinated.

**Conclusion:**

The protective effect of vaccination on morbidity was similar in those with heterologous vaccination as well as those who took

## Introduction

In December 2019, an acute respiratory diease caused by the novel coronavirus (SARS-CoV-2) emerged in China, later referred to as Coronavirus disease (COVID-19)^1^. The disease rapidly spread throughout the world, and by March 2020 the World Health Organization (WHO) declared it a pandemic^1^,^2^. It has since had a heavy burden in terms of morbidity and mortality, and as of the 17th May 2023, over 766 million cumulative cases and 6 million cumulative deaths had been reported worldwide^3^. In Africa, it stood at 13 million cases for 258 thousand deaths, while in Cameroon, we had over 125 thousand cses for close to 2 thousand deaths^3^. Experts point out that immunity through vaccination is crucial to ending the current pandemic^4^, with vaccination rates over 60% needed to achieve herd immunity^5^,^6^. Fortunately, several vaccines where developed in emergency in order to curb the spread and prevent progression to severe disease and death, with 10 vaccines approved by WHO as of the 8th April 2022 after meeting the necessary criteria for safety and efficacy^7^. Mass vaccination programs started in early December 2020 globally and in April 2021 in Cameroon^8^,^9^. That notwithstanding, vaccine trackers as of the 7th May 2023, showed 64.3% of the global population fully vaccinated for 30.6% in Africa and only 11.3% in Cameroon^3^.

With most COVID-19 vaccines requiring at least 2 doses given 3 to 12 weeks apart for complete protection^10^, and with the high demand for vaccines worldwide, the vaccine received during the first dose is not always available at the time for the second dose due to logistical bottlenecks of the COVID-19 vaccine supply chain, especially in low-income countries^10^. In his line, heterologous COVID-19 vaccination could facilitate mass vaccination programs^11^. However, despite WHO reporting it is safe and effective to mix and match different COVID-19 vaccines^7^, unwillingness to complete vaccination with a vaccine of a different type from that started remains a barrier, with one reason being insufficient data on benefits and safety of heterologous vaccination^12^.

To the best of our knowledge, very few studies have been done in our context to assess the effect of heterologous COVID-19 vaccination on morbidity and mortality. This study aimed at assessing the effectiveness of heterologous COVID-19 vaccination in reducing morbidity and mortality, which will help increase our knowledge on the effects of mixing various vaccines, hence, encourage more vaccination.

## Materials and methods

This was a 7 months hospital based retrospective cohort study, running from the 18th August 2021 to 28th February 2022. The study setting was the COVID-19. Treatment Centre of the Bamenda Regional Hospital, which is a tertiary referral hospital in the North West Region of Cameroon.

Ethical clearance was obtained from the Institutional Review Board (IRB) of the Faculty of Health Sciences of the University of Bamenda and administrative authorizations to carry out the research were gotten from the North West Regional Delegation of Public Health and from the Director of the Bamenda Regional Hospital.

Files of all patients aged ≥ 18 years managed by the said treatment centre within the study period with confirmed COVID-19 where included, after excluding files of fully vaccinated with a single dose regimen (Jannsen), files of partially vaccinated, and files with incomplete data on essential elements (age, vaccination status, SpO2, outcome).

A consecutive non probability sampling method was used, with the inclusion of all files meeting our selection criteria. However, a minimum sample size of 84 was required for enough statistical power and narrow margin of error of 5% using Cochran's formula, with expected prevalence of complete vaccination of 5.8% gotten from a study in Cameroon, done by chiabi et al in 2023^13^. We presented the necessary authorizations together with ethical clearance to the staff of the COVID-19 treatment centre in order to gain access to the patient's records. We then proceeded to retrieve all available files upon which we applied our selection criteria.

The files retained after application of selection criteria were exploited as follows; data was extracted using pre-designed questionnaires, with study variables being: age and gender as socio-demographic parameters, COVID-19 vaccination status as fully vaccinated or unvaccinated. When fully vaccinated, we went further to check if it was by heterologous or homologous vaccination. Other variables concerned presence or absence of co-morbidities, clinical presentation, clinical staging as asymptomatic, mild, moderate or severe disease), and finally, outcome which could be survived or death. We defined COVID-19 patient as Participant with positive RT-PCR (polymerase chain reaction) and/or a rapid antigenic test (RDT) for COVID-19; Fully/Completely vaccinated: Participants who received 2 doses of vaccine other than Johnson & Johnson (ie. Sinovac, Vaxzevria, Spikevax, Comirnaty/BioNTech vaccine), be it heterologous or homologous; Heterologous vaccination: Participants who received 2 doses of a COVID-19 vaccine (Johnson & Johnson excluded), where the second dose vaccine was different from the first dose in a 2-dose series (ie. Sinovac, Vaxzevria, Spikevax, Comirnaty/BioNTech vaccine); Homologous vaccination: Considered after receiving the second dose, which was the same type as that of the first dose in a 2-dose series (Sinovac, Vaxzevria, Spikevax, Comirnaty/ BioNTech vaccine); Unvaccinated: Did not receive any COVID-19 vaccine. Comorbidity was considered for patients with at least one of the following chronic diseases: hypertension, diabetes melitus, asthma, HIV, heart failure, renal failure and sickle cell disease, just to name a few.

Following data collection, data was transferred to Microsoft office excel 2016 for coding and later analysed using the statistical software SPSS (Statistical Package for the Social sciences) version 26, with statistical significance set at a p-value <0.05 at 95% confidence intervals. Association between vaccination status and disease severity as well as mortality was assessed by calculating odds ratios, with multivariate logistic regression used to control for confounders.

## Results

**Figure 1:**
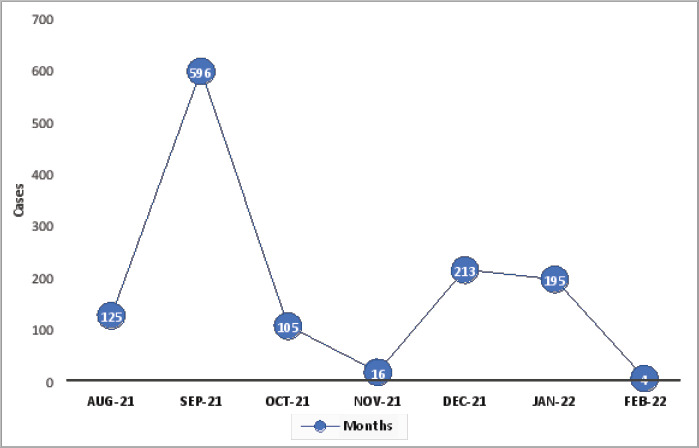
Temporal distribution of COVID-19 cases (N=1254)

Temporal distribution COVID-19 cases was highest in September 2021 with 596 cases (47.5%), followed by 213 (17.0%) in December 2021 and 195 (15.6%) in January 2022 (Figure 1). Throughout our study period, 1917 records of patients managed by the COVID-19 treatment centre were retrieved. We first excluded 218 patients < 18 years of age, 310 for having incomplete data on our important variables, 76 for being partially vaccinated (received only 1 dose of a 2-dose vaccine) and 59 vaccinated with a single dose Johnson & Johnson vaccine, leaving us with 1254 records which we included and analysed. A ttal 24 out of the 1254 (1.9%) of patients in the treatment centre were fully vaccinated (with 2 doses of multiple dose regimen).

Of our 1254 participants, 749 (59.7%) were females. The age of participants ranged from 18 to 100 years, with a mean of 50.1 (± 19.6) years; 453 (36.1%) had comorbidities; 327 (72.2%) with hypertension, followed by 128 (30.5%) with diabetes mellitus and 45 (9.9%) with HIV. Moreover, the mild form of the disease was the most common clinical stage (475; 37.9%).

Of the 24 completely vaccinated patients, 7 (29.2%) had received heterologous vaccines while 17 (70.8%) took homologous vaccines. Among those who took heterologous vaccines, majority were females (5; 71.4%), aged ≤ 60 years (6; 85.7%) and had no comorbidities (6; 85.7%). Regarding the vaccines, only Sinovac and/or Vaxzevria were administered (in the 24 who were completely vaccinated with multiple vaccines doses).

Overall, 721 (57.5%) had moderate to severe forms of the disease, with 713 (98.9%) being unvaccinated, 417 (57.8%) of female gender, 383 (53.1%) aged greater than 60 years and 387 (53.7%) had at least a comorbidity. Among the factors analysed, being fully vaccinated, regardless of homologous or heterologous vaccination was found to significantly decrease the odds of getting severe disease (aOR =0.63; CI95 (0.24-0.71); p=0.037). However, heterologous vaccination did not influence odds of severe disease as compared to those who received homologous vaccines (aOR =0.64; CI95 (0.05-8.02); p=0.729). On the other hand, being aged > 60 years (aOR =8.57; CI95 (6.08-12.09); p=0.000) and having comorbidities (aOR =6.36; CI95 (4.62-8.74); p=0.000) increased the odds of getting severe disease. (Table I).

**Table I:** Factors related to disease severity (N =1254)

Variables	Severe^a^	Non severe^b^	OR^c^ (95% CI^d^)	P-value	Multivariate logistic regression	
	
	Number 721(%)	Number 533(%)			aOR^e^ (95% CI^d^)	P-value
**Vaccination status**						
Not vaccinated	713(98.9)	517(97.0)	Reference		Reference	
Fully vaccinated	8(1.1)	16(3.0)	0.36 (0.15-0.85)	***0.020***	0.63 (0.24-0.71)	***0.037***
Homologous vaccination	6(0.8)	11(2.1)	Reference		Reference	
Heterologous vaccination	2(0.3)	5(0.9)	0.73 (0.09-5.00)	0.751	0.64 (0.05-8.02)	0.729
**Sex**						
Female	417(57.8)	332(62.3)	Reference		Reference	
Male	304(42.2)	201(37.7)	1.20 (0.96-1.51)	0.112	1.26 (0.96-1.67)	0.101
**Age (years)**						
≤ 60	338(46.9)	484(90.8)	Reference		Reference	
> 60	383(53.1)	49(9.2)	11.19 (8.06-15.54)	***0.000***	8.57 (6.08-12.09)	***0.000***
**Comorbidities**						
Absent	334(46.3)	457(87.6)	Reference		Reference	
Present	387(53.7)	66(12.4)	8.20 (6.90-11.03)	***0.000***	6.36 (4.62-8.74)	***0.000***

a Patients with moderate to severe form

b Patients with asymptomatic to mild form

c Odds ratio

d Confidence interval

e Adjusted odds ratio

Of the 721 with moderate to severe disease, 135 (18,7%) died, with none fully vaccinated. Moreover, 78 (57.8%) were males, 119 (88.1%) aged greater than 60 years, and 85 (63.0%) had comorbidities. With no fully vaccinated among the death, odds ratios could not be calculated for vaccination status relative to mortality. However, being of male gender (aOR=2.00; CI95 (1.34-3.00); p=0.001) and age > 60 years (aOR=8.42; CI95 (4.84-14.63); p=0.000) were found to significantly increase the odds of mortality. (Table II).

**Table II:** Factors related to mortality in patients with moderate to severe disease (N= 721)

Variables	Died	Survived	OR (95% CI)	P-value	Multivariate logistic regression	
	
	Number 135 (%)	Number 586 (%)			aOR (95% CI)	P-value
**Vaccination status**						
Not vaccinated	135(100)	578(98.6)	Reference		Reference	
Fully vaccinated	0(0.0)	8(1.4)	**Undefined**		**Undefined**	
**Sex**						
Female	57(42.2)	360(61.4)	Reference		Reference	
Male	78(57.8)	226(38.6)	2.18 (1.49-3.19)	***0.000***	2.00 (1.34-3.00)	***0.001***
**Age (years)**						
≤ 60	16(11.9)	322(54.9)	Reference		Reference	
> 60	119(88.1)	264(45.1)	9.07 (5.25-15.67)	***0.000***	8.42 (4.84-14.63)	***0.000***
**Comorbidities**						
Absent	50(37.0)	284(48.5)	Reference		Reference	
Present	85(63.0)	302(51.5)	1.60 (1.09-2.35)	***0.017***	1.23 (0.81-1.86)	0.332

## Discussion

This study aimed at assessing the effect of heterologous COVID-19 vaccination on morbidity as well as mortality in the BRH, Cameroon.

The vaccination rate for fully vaccinated was 1.9%. This was lower than the findings of Chiabi et al in Cameroon in 2023 which had 5.8% fully vaccinated 13, and far lower than 16% fully vaccinated found in India, in 2021 by Muthukrishnan et al 14; and equally less than 8% fully vaccinated found in the US by Scobie et al in 2021^15^. This difference could be explained by the fact that our study excluded patients fully vaccinated with Johnson & Johnson vaccine. Moreover, higher vaccine hesitancy is reported in sub-Saharan Africa compared to high vaccine acceptance in Asia and in the US^16^.

Being fully vaccinated was found to significantly decrease the odds of getting severe disease. This was in accordance with the findings of Chiabi et al in Cameroon in 2023^13^, as well as results of Macchia et al in Argentina, 2021 where the risk of getting severe disease was decreased by being fully vaccinated^17^. Heterologous vaccination did not significantly decrease odds of severe disease when compared to homologous vaccination. By contrast, in another study from the UK, heterologous COVID-19 vaccination was significantly more protective against COVID-19 and hospitalisation^11^. Heterologous vaccination is thought to induce a robust and sustained elevation of specific immunity as it combines induction of antibodies and CD4+ T-cells from protein subunit vaccines, stimulates cellular immunity from DNA vaccines, and enhancement of cellular and humoral immunity from viral vector vaccines^10^. The disparity of findings from our study may be partly explained by the number of doses of vaccine received (max two), with only one inactivated vaccine (Siopharm/Sinovac) or one viral vector vaccine (AstraZeneca/Vaxzevria) received. However, in another observational study conducted on university staff from North-Eastern Italy, protection from symptomatic SARS-CoV-2 infection increased with higher number of doses of COVID-19 vaccine, regardless of heterologous or homologous booster dose^18^. Nevertheless, with Omicron the main barriers against the spread of occupational asymptomatic infections in highly vaccinated workforce remained non-pharmaceutical risk reduction measures^19^. No deaths were observed among fully vaccinated patients, confirming the protective effect of complete vaccination cycle against COVID-19 associated mortality.

## Limitations

As all retrospective studies, some files had missing data on important variables. Full vaccination coverage was low (1.9%), which likely impacted the significance of some statistical estimates. Furthermore, we did not have information to distinguish deaths due to COVID-19 from those with COVID-19.

## Conclusion

Full vaccination coverage was as low as 1.9% in the present study. The odds of getting severe disease were reduced for fully vaccinated patients compared to the unvaccinated, with this protective effect similar in those with homologous and heterologous vaccination. Regarding mortality, the absence of any deaths among fully vaccinated confirmed the protective effect of complete vaccination cycle against COVID-19 associated mortality.

## References

[1] Wang D, Hu B, Hu C, Zhu F, Liu X, Zhang J (2020). Clinical Characteristics of 138 Hospitalized Patients With 2019 Novel Coronavirus–Infected Pneumonia in Wuhan, China. JAMA.

[2] Alyami MH, Naser AY, Orabi MAA, Alwafi H, Alyami HS (2020). Epidemiology of COVID-19 in the Kingdom of Saudi Arabia: An Ecological Study. Front Public Health.

[3] Mathieu E, Ritchie H, Rodés-Guirao L, Appel C, Giattino C, Hasell J (2020). Coronavirus Pandemic (COVID-19). Our World in Data.

[4] Cheng H, Peng Z, Si S, Alifu X, Zhou H, Chi P (2022). Immunogenicity and Safety of Homologous and Heterologous Prime–Boost Immunization with COVID-19 Vaccine: Systematic Review and Meta-Analysis. Vaccines (Basel).

[5] Cascini F, Pantovic A, Al-Ajlouni Y, Failla G, Ricciardi W (2021). Attitudes, acceptance and hesitancy among the general population worldwide to receive the COVID-19 vaccines and their contributing factors. E Clinical Medicine.

[6] Randolph HE, Barreiro LB (2020). Herd Immunity: Understanding COVID-19. Immunity.

[7] (2022). COVID-19 Vaccines Advice.

[8] (2021). Coronavirus disease (COVID-19): Vaccines.

[9] (2021). MINSANTE| Covid-19: La vaccination a commencé.

[10] Garg I, Sheikh AB, Pal S, Shekhar R (2022). Mix-and-Match COVID-19 Vaccinations (Heterologous Boost): A Review. Infect Dis Rep.

[11] Liu X, Shaw RH, Stuart ASV, Greenland M, Aley PK, Andrews NJ (2021). Safety and immunogenicity of heterologous versus homologous prime-boost schedules with an adenoviral vectored and mRNA COVID-19 vaccine (Com-COV): a single-blind, randomised, non-inferiority trial. Lancet.

[12] (2022). Five things to know about: Mixing and matching coronavirus vaccines | Research and Innovation.

[13] Chiabi A, Unji PK, Mambap Tatang A, Angwafor S, Niba LL, Porro Adji MJ (2023). COVID-19 Vaccination: Impact on Disease Severity and Mortality in an African Setting. Academic Journal of Health Sciences: Medicina Balear.

[14] Muthukrishnan J, Vardhan V, Mangalesh S, Koley M, Shankar S, Yadav AK (2021). Vaccination status and COVID-19 related mortality. Med J Armed Forces India.

[15] Scobie HM, Johnson AG, Suthar AB, Severson R, Alden NB, Balter S (2021). Monitoring Incidence of COVID-19 Cases, Hospitalizations, and Deaths, by Vaccination Status in 13 U.S. Jurisdictions. Morb Mortal Wkly Rep.

[16] Ngwewondo A, Nkengazong L, Ambe LA, Ebogo JT, Mba FM, Goni HO (2020). Knowledge, attitudes, practices of/towards COVID 19 preventive measures and symptoms. PLoS Negl Trop Dis.

[17] Macchia A, Ferrante D, Angeleri P, Biscayart C, Mariani J, Esteban S (2021). Evaluation of a COVID-19 Vaccine Campaign and SARS-CoV-2 Infection and Mortality Among Adults Aged 60 Years And Older in a Middle-Income Country. JAMA Netw Open.

[18] Cegolon L, Negro C, Pesce M, Filon FL (2023). COVID-19 Incidence and Vaccine Effectiveness in University Staff, 1 March 2020–2 April 2022. Vaccines (Basel).

[19] Cegolon L, Ronchese F, Ricci F, Negro C, Larese-Filon F (2022). SARS-CoV-2 Infection in Health Care Workers of Trieste (North-Eastern Italy), 1 October 2020–7 February 2022: Occupational Risk and the Impact of the Omicron Variant. Viruses.

